# The Economic Burden of Malaria: Revisiting the Evidence

**DOI:** 10.4269/ajtmh.19-0386

**Published:** 2019-10-14

**Authors:** Nayantara Sarma, Edith Patouillard, Richard E. Cibulskis, Jean-Louis Arcand

**Affiliations:** 1 Department of International Economics, The Graduate Institute, Geneva, Switzerland;; 2 World Health Organization, Geneva, Switzerland

## Abstract

A portion of the economics literature has long debated about the relative importance of historical, institutional, geographical, and health determinants of economic growth. In 2001, Gallup and Sachs quantified the association between malaria and the level and growth of per capita income over the period 1965–1995 in a cross-country regression framework. We took a contemporary look at Gallup and Sachs’ seminal work in the context of significant progress in malaria control achieved globally since 2000. Focusing on the period 2000–2017, we used the latest data available on malaria case incidence and other determinants of economic growth, as well as macro-econometric methods that are now the professional norm. In our preferred specification using a fixed-effects model, a 10% decrease in malaria incidence was associated with an increase in income per capita of nearly 0.3% on average and a 0.11 percentage point faster per capita growth per annum. Greater average income gains were expected among higher burden countries and those with lower income. Growth of industries with the same level of labor intensity was found to be significantly slower in countries with higher malaria incidence. To analyze the causal impact of malaria on economic outcomes, we used malaria treatment failure and pyrethroid-only insecticide resistance as exogeneous instruments in two-stage least squares estimations. Despite several methodological challenges, as expected in these types of analyses, our findings confirm the intrinsic link between malaria and economic growth and underscore the importance of malaria control in the agenda for sustainable development.

## INTRODUCTION

Investing in health has been considered a means to achieve economic growth and reduce poverty since the second half of the 20th century. Until then, economic thinking was about a one-way relationship between wealth and health in terms of wealth being required to achieve health.^1–6^ This linear wealth-to-health link was weakened by several econometric studies providing evidence that health is a significant determinant of growth.^4,7^ In 2001, the WHO Commission on Macroeconomics and Health underscored the importance of health as an instrument for economic development and poverty reduction.^8^ Bloom and Canning described the process of cumulative causality where health improvements promote economic growth, which in turn promotes health. Healthier populations are more productive at work and learn more at school, contributing to increased current and future earnings and savings. Associated savings in health-care spending is hypothesized to increase investment in physical and human capital and attract higher foreign investments. Higher income for individuals or countries improves health through different channels, from better nutrition to better public health infrastructure.^9^ Furthermore, as per the classical Grossman model of health demand, if individuals expect a longer life, their savings and investment in human capital will be greater.^10^ The mutual reinforcement between health and wealth is also recognized to exist in reverse, whereby sick people are more likely to become poor and those who are poor are more vulnerable to disease.^11^

Although empirically there is evidence on the strong correlation between health and income both across and within countries over time, the literature has long debated on the magnitude of the effects of other factors that simultaneously influence health outcomes and wealth, notably institutional quality.^6,9,12,13^ Specifically, a portion of the empirical growth economics has long debated about the relative importance of potential determinants of economic growth. In 2001, Gallup and Sachs quantified the association between malaria and the level and growth of per capita income over the period 1965–1995. In a cross-country regression framework controlling for historical, geographical, social, economic, and institutional country characteristics, they found that malaria-endemic countries displayed, ceteris paribus, per capita income levels 70% lower than those of nonendemic countries and that a 10% reduction in their malaria exposure index was associated with a 0.26 percentage point increase in annual per capita growth rates.^14^ By contrast, Acemoglu, Johnson, and Robinson argued that malaria was not the main determinant of economic performance, instead concluding on the central role of institutions in cross-country growth differences.^15^ Despite this debate, results from the Gallup and Sachs study are still widely cited to support the case for investing in malaria control and elimination in the context of the Sustainable Development Goals.^16–25^

Since the publication of these studies, significant progress has been made in the fight against malaria and estimation methods have evolved. Core malaria control interventions have reached unprecedented coverage levels, and this progress contributed to reduce malaria case incidence rate by an estimated 37% globally over the period 2000–2015 and by 18% over the more recent 2010–2017 period.^26^ The availability of data and methodologies for estimating malaria-burden estimates has also improved. Although some data on malaria incidence were available from the WHO at the time of the Gallup and Sachs study, the authors preferred using a malaria exposure index, defined as the product of the land area subject to malaria and the fraction of malaria cases attributable to *Plasmodium falciparum* malaria.^14^ Since then, malaria case incidence measures have been standardized.^27,28^ New econometric approaches exploiting panel data structures have also become the norm.^29,30^

Herein, we take a contemporary look at the seminal work of Gallup and Sachs.^14^ We focus on the period 2000–2017 and draw on the vastly improved and more recent data available on malaria case incidence and other determinants of economic growth as well as the much higher econometric standards that are now the professional norm. Then, we make an attempt at a causal analysis of malaria on economic growth using two instrumental variables (IVs), namely, antimalarial treatment failure and resistance of malaria mosquitos to pyrethroid-only insecticides. We supplement this study with a sectoral analysis testing the hypothesis that industry sectors that are more labor intensive will have slower growth rates in countries that have higher malaria incidence. Evidence suggests that in addition to the implications on the total size of the economy, malaria is associated with reduced labor productivity and supply.^31–37^ To explore this further, we use a common approach in macro-econometric modeling recently applied to the health sector to quantify the argument that malaria case incidence affects economic growth through labor productivity.^38,39^

## MATERIAL AND METHODS

### Methods.

For our contemporary take at the seminal work of Gallup and Sachs, our specification of the gross domestic product per capita (GDPpc) purchasing power parity (PPP) regressions in level can be written as follows:

yit = xit α + mitβ + uit, (1)

where *i* indexes countries, *t* refers to the year, and *y*_*it*_ refers to log GDPpc PPP; *m*_*it*_ is the log malaria case incidence per 1,000 population, which is transformed using the standard inverse hyperbolic sine transformation IHS(*y*_*i*_) = log[*y*_*i*_ + (*y*_*i*_2 +1)^1/2^] to reduce the effect of outliers and account for not being able to take the log of zero; *x*_*it*_ is a matrix of covariates relating to the historical, geographical, and socioeconomic characteristics of each country; finally, *u*_*it*_ is the error term that can be decomposed into a standard two-way fixed-effects specification posed as follows:^40^

$$u_{\mathrm{it}}{\;=\lambda }_{i}{\;+µ}_{t}{\;+\varepsilon }_{\mathrm{it}},$$ (1')

where λ_*i*_ and µ_*t*_ are country- and year-specific unobservables, respectively, and ε_*it*_ is the error term that represents idiosyncratic shocks occurring at the country–year level.

Our growth analysis is conducted on 5-year averages so as to smooth data on the GDPpc PPP growth rate that often vary widely year-on-year.^41^ Our specification can be written as follows:

$$\Delta y_{ip}\;=\;y_{ip-1}\alpha \;+\;m_{ip-1}\beta \;+\;\Delta m_{ip}\beta \;+\;x_{ip}\gamma \;+\;u_{ip},$$ (2)

where *i* indexes countries and *p* is the time period; Δ*y*_*ip*_ refers to the 5-year average annual growth of GDPpc PPP; *y*_*ip*−1_ is the lagged level of GDPpc PPP in log; *m*_*ip*−1_ is the 5-year average log malaria incidence per 1,000 population in the time period *p*−1; *m*_*ip*_ is the 5-year average log change of malaria incidence in the time period *p*; and *x*_*ip*−1_ is a matrix of covariates relating to institutional and socioeconomic characteristics of each country. Covariates referring to a country’s historical and geographical characteristics are time invariant and, therefore, accounted for by the country-specific fixed effects. The error term decomposition described by equation (1’) applies for equation (2) with 5-year time periods.

Equations (1) and (2) are estimated using three methods. The first method uses the Ordinary Least Squares method (OLS) to explore the association between malaria case incidence and GDPpc PPP across countries at specific points in time.^14^ The second method transforms the equations into deviations with respect to country-specific means (“within” transformation in a fixed-effects model) to eliminate all factors that do not change over time for each country (λ_*i*_). The third method is the two-stage least squares method (2SLS) to account for potential endogeneity, that is, correlation of the malaria incidence variables with unobserved or omitted terms included in the error term *ε*_*it*_. This third method requires the identification of an IV, a method developed to control for unmeasured confounders in observational studies.^42^ We explored the validity of two IV candidates. One is antimalarial drug efficacy measured by the percentage of malaria patients with treatment failure, calculated across study protocols and drug types at the country–year level.^43,44^ Treatment failure is defined as the inability to clear parasites from a patient’s blood or to prevent their recrudescence after the administration of an antimalarial, regardless of whether clinical symptoms are resolved.^45^ Another candidate is resistance of mosquitos to insecticides measured by the percentage of studies reporting pyrethroid-only resistance status as confirmed, possible, and susceptible, at the country–year level.^44,46^

To be valid, an instrument needs to satisfy three conditions: relevance (it affects malaria incidence), exclusion (its affects GDPpc only indirectly through its effects on malaria case incidence), and independence (of unmeasured confounding).^42,47^ The relevance assumption is verified empirically from first-stage regression of the 2SLS method using the *F*-statistics (see the Results section). As a rule of thumb, an instrument is considered weak if the *F*-statistics is less than 10.^48^ The exclusion and independence assumptions cannot be verified from the data, so we applied subject–matter intuition. It seems reasonable to assume that antimalarial treatment failure and resistance of malaria mosquitos to pyrethroid-only insecticides affect GDPpc only through populations or areas at risk of malaria transmission and, conditional on the covariates, affecting GDPpc solely via malaria incidence. In addition, the use of panel data in a fixed-effects model is assumed to mitigate the risk of the IV being associated with unobserved variables also affecting GDPpc. To exploit both instruments, treatment failure and insecticide resistance, we supplemented the panel IV analysis by a pooled IV approach, where we use only those observations for which data on both candidates are available. Because of the reduced sample size, we cannot include country-specific effects in the pooled-IV approach. Finally, for both panel IV and pooled-IV methods, we conducted the analysis allowing standard errors to be correlated among countries within a subregion to reflect the relevance of our instruments at a subregional level, instead of their containment within country borders. The analyses are also conducted with country-level clustering of standard errors for comparative purposes.

The specification used in our sectoral analysis can be written as follows:

gijt = lijt α+(lijt×mit) β+uijt, (3)

$$\text{where}\;u_{ijt}\;=\;\rho_{it}\;+\;\kappa_{ij}\;+\;\tau_{jt}\;+\;\varepsilon_{ijt},$$ (3')

and *i* indexes countries, *t* refers to year, *j* indicates the industry sector, *g*_*ijt*_ is the annual growth rate of the value added of industry *j*, *l*_*ijt*_ is the share of the wage bill in the total value added of industry *j* in country *i* and year *t*, *m*_*it*_ is the log of malaria case incidence in country *i* and year *t* and ρ_*it*_, κ_*ij*_, and τ_*jt*_ are the country–year-, country–industry-, and industry–year-specific effects, respectively, which control for the effect of other factors varying at those levels and that may potentially affect growth. We include one way effects for estimations where the full set of two-way effects are not included. The main coefficient of interest in equation (3) is β, which is essentially the cross partial derivative of the dependent variable *g*_*ijt*_ with respect to *l*_*ijt*_ and then with respect to *m*_*it*_*.* In other words, β denotes the effect of a marginally higher labor share on value-added industrial growth for a marginally higher incidence of malaria. Because malaria incidence measures vary only at the country–year level, the inclusion of the full set of fixed effects wipes out the effect of malaria incidence on its own, and we are left with the interaction term and direct effect of the labor share, both of which vary at the more granular industry–country–year level. The point estimate β allows us to make, and quantify, the plausible argument that malaria incidence affects economic growth through labor productivity. This “difference-in-differences” specification has been common in macro-econometric modeling, notably on the effect of financial depth on economic growth (financial depth varies at the country–year level, whereas different industrial sectors display different levels of dependence on external finance).^38^ More recently, this type of specification has been applied to the impact of health sector workforce employment on economic growth.^39^

### Materials.

We used data from 180 countries over the period 2000–2017. Although our objective is to keep with the spirit of the Gallup and Sachs study, it is not possible to faithfully replicate it using updated data because a number of explanatory variables are no longer maintained or have been superseded by alternative measures. We used malaria case incidence per 1,000 population at the country–year level, calculated as the ratio of the estimated number of malaria cases and United Nations total population estimates multiplied by 1,000.^27,49^ We used total population data instead of population at risk of malaria estimates to match incidence and GDPpc data. In addition, the global burden of malaria lies mostly in countries that have a total population at risk of malaria equal to their total population estimates. And, in countries that have reached malaria elimination or malaria-free status over the period 2000–2017, the use of total population as the denominator allows us to capture the decline in case incidence that would not be fully captured with a decreasing population at risk of malaria over the study period. Data on the level of GDPpc PPP and the annual growth therein are sourced from the World Bank’s World Development Indicators (WDI).^50^ Data on trade openness, which is proxied by the value of trade expressed in terms of percentage of GDP, and data on life expectancy at birth are also from the WDI. We used Barro–Lee educational attainment data, expressed in terms of the average number of years of schooling in the population older than 15 years.^51^ In OLS specifications where covariates include whether a country is landlocked or a former colony, data come from the GeoDist database of the CEPII.^52^ Data on the quality of institutions are captured by the Rule of Law estimate from the World Bank’s Worldwide Governance Indicators project.^53^ When exploring the causal effect of malaria on GDPpc PPP, we used WHO’s data on antimalarial treatment failure and insecticide resistance as instruments and restricted the analysis to the period for which data are available. Finally, to explore the possible mechanism through which malaria and sectoral economic performance interact, data on the manufacturing industry’s value-added and share of labor costs of the total value added are sourced from the United Nations Industrial Development Organization for 96 countries over the period 2000–2010.^54^ These data are available for 23 manufacturing industries at the 2-digit level of the International Standard Industrial Classification of All Economic Activities Revision 3. Table 1 shows the variables and their source. Tables 2 and 3 summarize the data.

**Table 1:** Description of variables used in the models

Variable	Description	Source
Economic variables		
Log GDPpc PPP	Log of GDP per capita in PPP international dollars 2011	50
Log GDPpc PPP growth rate	Log of GDP per capita PPP annual growth rate	50
Industry growth rate	Annual growth rate of industry (2-digit Industrial Classification of All Economic Activities) value added	54
Labor share	Ratio of the wage bill over total industry value added	54
Malaria-related variable		
Log malaria incidence	Log of malaria case incidence per 1,000 population	27
Log annual change incidence	Log of annual change in malaria case incidence per 1,000 population	27
Antimalarial treatment failure	Percentage of patients with malaria treatment failure (per protocol)	27,44
Insecticide resistance	Percentage of studies with resistance status classified as confirmed, possible, and susceptible	27,44
Institutional, socioeconomic, and geographical variables		
Rule of Law	Country-specific annual score on aggregate Rule of Law indicator measuring the extent to which agents have confidence in and abide by the rules of society	50
Life expectancy at birth	Country-specific annual average number of years a newborn is expected to live	50
Years of schooling	Average number of years of schooling in population aged 15+ years	51
Colony	Dummy variable for whether the country was a colony	52
Landlocked	Dummy variable for whether the country is landlocked	52

GDPpc = gross domestic product per capita; PPP = purchasing power parity.

**Table 2:** Summary statistics

	Count	Mean	SD	Minimum	Maximum	No. of countries
All countries						
*** ***Economic variables						
*** ***GDPpc PPP over the period 2000–2017	2,948	16,730.75	19,101.90	545.69	129,349.92	180
*** ***GDPpc PPP average growth rate between 2000 and 2017	155	0.02	0.02	−0.02	0.09	155
*** ***GDPpc PPP average annual growth rate over the period 2000–2017	2,778	0.02	0.05	−0.97	0.80	180
*** ***2-digit industry value–added growth rate	11,829	0.08	0.41	−5.92	6.72	
*** ***2-digit industry labor share	11,829	0.40	0.18	0	1	
*** ***Malaria-related variables						
*** ***Malaria incidence	2,948	58.18	128.82	0.00	736.44	180
*** ***Log malaria incidence	2,948	1.63	2.49	0.00	7.29	180
*** ***Log annual change in malaria incidence	2,780	−0.27	1.58	−6.29	6.12	180
*** ***Institutional, socioeconomic, and geographical variables						
*** ***Rule of Law	2,948	−0.05	0.98	−2.01	2.10	180
*** ***Years of schooling	400	7.87	2.86	1.08	13.18	136
*** ***Net secondary enrollment rate	1,516	71.37	24.36	3.23	99.91	159
*** ***Colony	2,908	0.89	0.31	0.00	1.00	177
*** ***Landlocked	2,908	0.20	0.40	0.00	1.00	177
*** ***Life expectancy at birth	2,730	69.50	9.10	38.70	83.98	178
SSA						
*** ***Economic variables						
*** ***GDPpc PPP over the period 2000–2017	726	4,513.97	6,061.35	545.69	40,015.82	45
*** ***GDPpc PPP average growth rate between 2000 and 2017	41	0.02	0.02	−0.02	0.06	41
*** ***GDPpc PPP average annual growth rate over the period 2000–2017	684	0.02	0.06	−0.78	0.25	45
*** ***2-digit industry value–added growth rate	1,000	0.07	0.43	−2.46	4.08	
*** ***2-digit industry labor share	1,000	0.37	0.22	0.00	1.00	
Malaria-related variables						
*** ***Malaria incidence	726	219.18	169.16	0.00	607.11	45
*** ***Log malaria incidence	726	5.05	2.27	0.00	7.10	45
*** ***Log annual change in malaria incidence	685	−0.85	2.77	−5.32	6.12	45
*** ***Institutional, socioeconomic, and geographical variables						
*** ***Rule of Law	726	−0.67	0.64	−2.01	1.08	45
*** ***Net secondary enrollment	259	33.74	19.58	3.23	88.58	39
*** ***Life expectancy at birth	685	57.19	6.71	38.70	74.39	45
Non-SSA countries						
*** ***Economic variables						
*** ***GDPpc PPP over the period 2000–2017	2,222	20,722.37	20,185.03	1,044.95	129,349.92	135
*** ***GDPpc PPP average growth rate between 2000 and 2017	114	0.03	0.02	−0.02	0.09	114
*** ***GDPpc PPP average annual growth rate over the period 2000–2017	2,094	0.02	0.05	−0.97	0.80	135
*** ***2-digit industry value–added growth rate	10,829	0.09	0.41	−5.92	6.73	
*** ***2-digit industry labor share	10,829	0.41	0.18	0.00	1.00	
*** ***Malaria-related variables						
*** ***Malaria incidence	2,222	5.57	37.90	0.00	736.44	135
*** ***Log malaria incidence	2,222	0.51	1.22	0.00	7.29	135
*** ***Log annual change in malaria incidence	2,095	−0.08	0.80	−6.29	6.00	135
*** ***Institutional, socioeconomic, and geographical variables						
*** ***Rule of Law	2,222	0.15	0.99	−1.92	2.10	135
*** ***Net secondary enrollment	1,257	79.12	16.88	15.62	99.91	120
*** ***Life expectancy at birth	2,045	73.63	5.27	56.64	83.98	133

GDPpc = gross domestic product per capita; PPP = purchasing power parity; SSA = sub-Saharan Africa.

Note: Summary statistics provided for data in the estimation sample. The largest sample includes 180 countries for an average of 16 years for each country.

**Table 3:** Summary statistics of insecticide resistance and antimalarial treatment failure

	Count	Mean	SD	No. of countries
Central Asia				
*** ***Antimalarial treatment failure (%)	–	–	–	
*** ***Confirmed insecticide resistance	3	0.00	0.00	1
*** ***Possible insecticide resistance	3	0.00	0.00	1
*** ***Insecticide susceptible	3	1.00	0.00	1
Eastern Asia				
*** ***Antimalarial treatment failure (%)	6	0.75	1.16	1
*** ***Confirmed insecticide resistance	11	0.87	0.16	1
*** ***Possible insecticide resistance	11	0.05	0.07	1
*** ***Insecticide susceptible	11	0.08	0.15	1
Latin America and the Caribbean				
*** ***Antimalarial treatment failure (%)	16	2.07	3.92	3
*** ***Confirmed insecticide resistance	18	0.32	0.33	5
*** ***Possible insecticide resistance	18	0.10	0.16	5
*** ***Insecticide susceptible	18	0.58	0.35	5
Melanesia				
*** ***Antimalarial treatment failure (%)	6	8.05	6.77	3
*** ***Confirmed insecticide resistance	8	0.00	0.00	2
*** ***Possible insecticide resistance	8	0.12	0.17	2
*** ***Insecticide susceptible	8	0.88	0.17	2
Northern Africa				
*** ***Antimalarial treatment failure (%)	8	3.44	3.04	1
*** ***Confirmed insecticide resistance	8	0.63	0.29	1
*** ***Possible insecticide resistance	8	0.12	0.09	1
*** ***Insecticide susceptible	8	0.25	0.31	1
Southeastern Asia				
*** ***Antimalarial treatment failure (%)	50	5.25	6.10	8
*** ***Confirmed insecticide resistance	56	0.10	0.15	9
*** ***Possible insecticide resistance	56	0.09	0.14	9
*** ***Insecticide susceptible	56	0.81	0.23	9
Southern Asia				
*** ***Antimalarial treatment failure (%)	33	0.60	1.01	7
*** ***Confirmed insecticide resistance	58	0.27	0.27	8
*** ***Possible insecticide resistance	58	0.17	0.19	8
*** ***Insecticide susceptible	58	0.56	0.33	8
Sub-Saharan Africa				
*** ***Antimalarial treatment failure (%)	88	1.70	2.05	25
*** ***Confirmed insecticide resistance	300	0.52	0.37	37
*** ***Possible insecticide resistance	300	0.14	0.18	37
*** ***Insecticide susceptible	300	0.34	0.37	37
Western Asia				
*** ***Antimalarial treatment failure (%)	4	1.37	1.62	1
*** ***Confirmed insecticide resistance	10	0.39	0.39	2
*** ***Possible insecticide resistance	10	0.23	0.19	2
*** ***Insecticide susceptible	10	0.38	0.40	2
All countries				
*** ***Antimalarial treatment failure (%)	211	2.61	4.10	49
*** ***Confirmed insecticide resistance	472	0.43	0.37	66
*** ***Possible insecticide resistance	472	0.13	0.18	66
*** ***Insecticide susceptible	472	0.44	0.39	66

## RESULTS

Using an OLS cross-sectional regression in the year 2000 and controlling for historical, institutional, geographic, and socioeconomic covariates, we find that a 10% reduction in malaria case incidence is associated with a 1.8% increase in the level of GDPpc PPP on average (Table 4, column 1). This is the result of our specification that is closest to the Gallup and Sachs simple cross-sectional model. Pooling observations for the period 2000–2017 does not change the elasticity by much: a 10% decrease in malaria case incidence is associated with an increase in GDPpc PPP of 1.9% on average (Table 4, column 2).

**Table 4:** OLS and within regressions of log GDPpc PPP on log malaria incidence

	OLS 2000 (1)	OLS pooled (2)	Within all (3)	Within all (4)	Within non-SSA (5)	Within pre-2014 (6)
Log malaria incidence	−0.183*** [−0.263, −0.102]	−0.192*** [−0.234, −0.151]	−0.032** [−0.062, −0.001]	−0.027* [−0.056, 0.001]	−0.049** [−0.091, −0.007]	−0.035*** [−0.061, −0.009]
Colony	0.517*** [0.373, 0.661]	−0.198** [−0.355, −0.042]	–	–	–	–
Landlocked	−0.185 [−0.504, 0.134]	−0.423*** [−0.562, −0.283]	–	–	–	–
Rule of Law	−0.420*** [−0.684, −0.155]	0.486*** [0.405, 0.566]	0.189*** [0.106, 0.273]	0.196*** [0.113, 0.279]	0.205*** [0.105, 0.304]	0.150*** [0.052, 0.248]
Trade per cent of GDP	0.002** [0.000, 0.004]	0.002*** [0.001, 0.003]	−0.000 [−0.001, 0.000]	−0.001** [−0.001, −0.000]	−0.001** [−0.002, −0.000]	−0.001** [−0.001, −0.000]
Years of schooling	0.066* [−0.007, 0.138]	0.080*** [0.041, 0.118]	–	–	–	–
Year trend	No	No	Yes	No	No	No
Year effects	No	No	No	Yes	Yes	Yes
R-squared	0.776	0.794	0.514	0.533	0.551	0.499
Number of observations	132	400	2,948	2,948	2,222	2,266
Number of countries	–	136	180	180	135	180

GDPpc = gross domestic product per capita; PPP = purchasing power parity.

Note: OLS 2000 (1) is the closest specification to the Gallup and Sachs simple cross-sectional model; 95% CIs in brackets next to coefficients; * *P* < 0.10, ** *P* < 0.05, *** *P* < 0.01; Huber–White robust standard errors used for OLS and clustered at the country level for within estimations.

**Figure 1.**
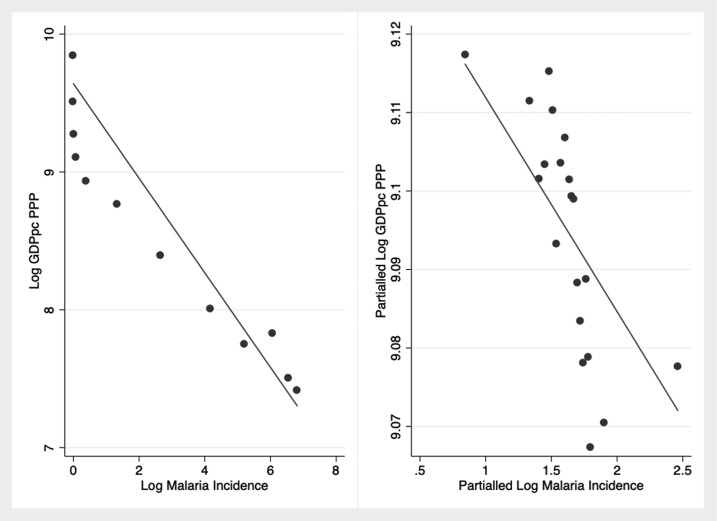
Association between malaria incidence and gross domestic product per capita (GDPpc) purchasing power parity (PPP) over the period 2000–2017. The figure on the left (*N* = 2,948) shows the simple binned correlation between GDPpc PPP and malaria case incidence. This relationship remains after both variables are purged of the effect of institutions, trade–GDP ratio, country, and time effects (right, *N* = 2,948). For visual clarity, the figures group observations into equally sized “bins” based on log malaria incidence and plot the mean log malaria incidence with the respective mean log GDPpc PPP.

**Figure 2.**
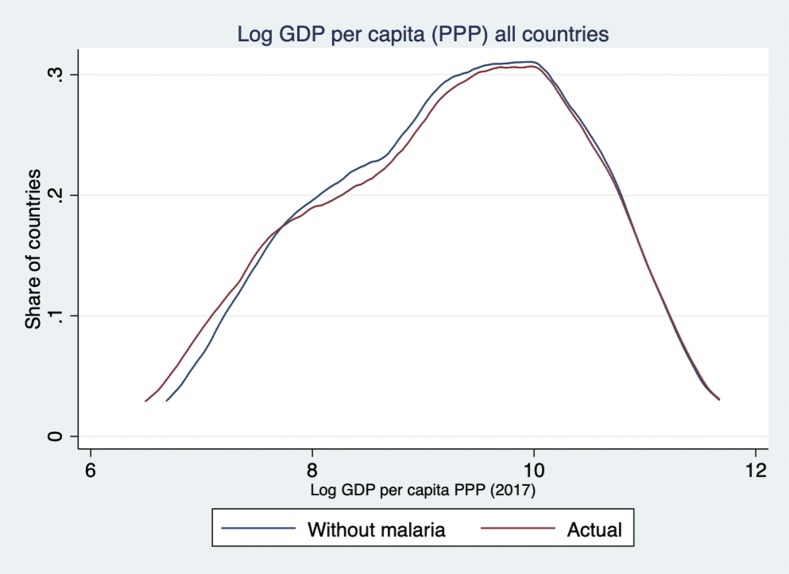
The world distribution of log gross domestic product per capita (GDPpc) purchasing power parity (PPP): actual (gray) and as predicted with a 100% reduction in malaria incidence (black). Simulation based on column 4 of Table 4. The vertical *y*-axis displays the proportion of countries. The horizontal *x*-axis displays log GDPpc PPP in 2017. The gray and black curves are continuous functions used to display the distribution of log GDPpc PPP across countries in 2017. Reading from the left to right: the gray curve shows that at 2017 malaria transmission levels, countries in the lowest 10th percentile of income had a log GDPpc PPP of 7.53 or less, equivalent to GDPpc PPP of 1,863; the black curve shows that without malaria, countries in the lowest 10th percentile of income would have a log GDPpc of 7.66 or less, equivalent to GDPpc PPP of 2,122. This indicates a rightward shift in log GDPpc PPP on the *x*-axis. Similarly, at 2017 transmission levels, 16.3% of countries had a log GDPpc PPP of 8 or below, equivalent to GDPpc PPP of 2,981 (gray curve). Assuming no malaria transmission in 2017, only 15.3% of countries would have a log GDPpc PPP of 8 or less. The crossing of the black and gray curves on the left side of the graph indicates a shift in the country mass, as low GDPpc PPP countries move to the right because of the economic gains associated with no malaria transmission. Finally, as expected, there is no change in the right side of the distribution as high GDPpc PPP countries have low or no malaria transmission, and thus, a change in transmission will not be associated with any economic gains from malaria elimination, according to our empirical model.

**Figure 3.**
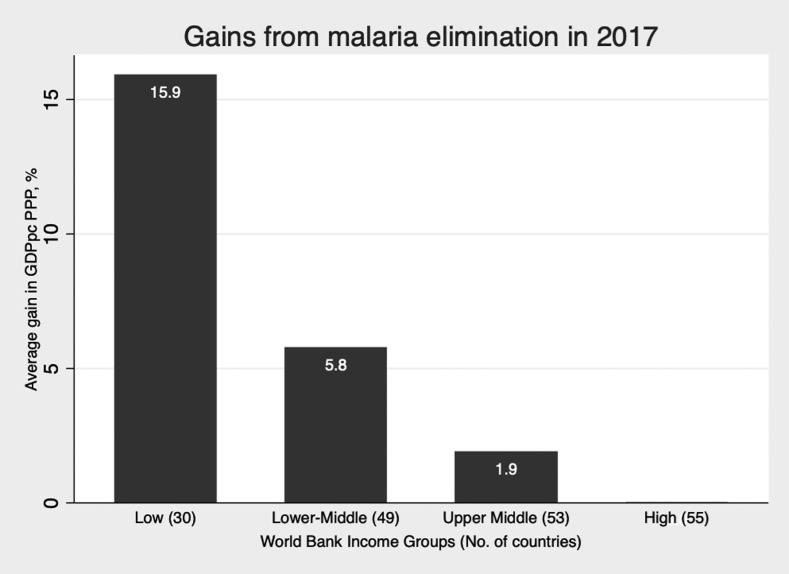
Average gross domestic product per capita (GDPpc) purchasing power parity (PPP) gain (%), by the World Bank income group in 2017.

**Figure 4.**
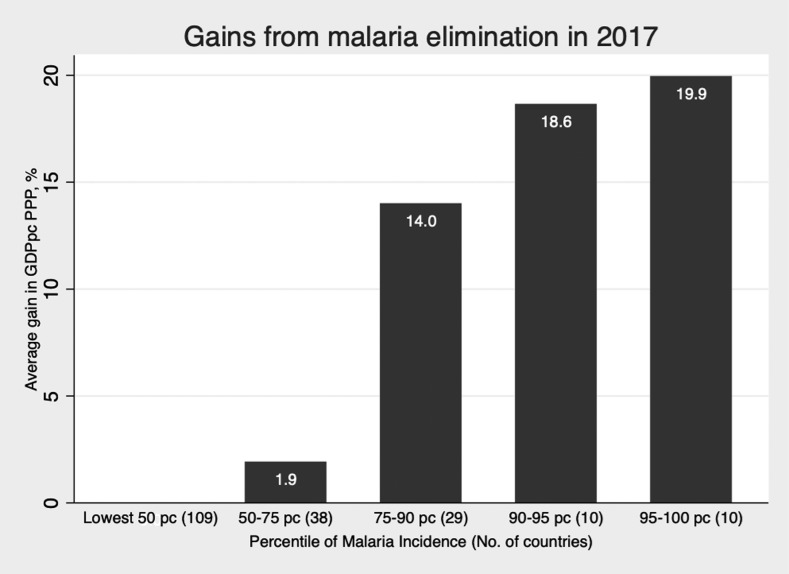
Average gross domestic product per capita (GDPpc) purchasing power parity (PPP) gain (%), by percentile of malaria incidence in 2017. Note: At the 50th percentile, that is, the median country has zero malaria incidence. Country at the 75th percentile has 6.5 cases per 1,000 population; at the 90th percentile, 194 cases per 1,000 population; and at the 95th percentile, 338 cases. Malaria incidence data are for the year 2017.

After introducing time-invariant country-specific effects and year-specific effects, a 10% reduction in malaria incidence is associated with a 0.27% increase in the level of GDPpc PPP on average (Table 4, column 4, Figure 1). The reduction in the size of the coefficient compared with the OLS method reflects how much of the variation in GDPpc PPP is explained by the fixed effects alone, that is, unobserved time-invariant heterogeneity within each country. These results are not driven by the sub-Saharan Africa (SSA) subsample, indicating that the relationship between malaria and economic growth holds in countries outside SSA as well. Excluding SSA countries where malaria incidence is the highest, a 10% reduction in incidence is associated with an increase in GDPpc PPP of nearly 0.5% on average (Table 4, column 5). Assuming constant elasticity between income and incidence, malaria eradication (defined as a 100% decrease in case incidence worldwide) would be associated with a rightward shift in the world probability distribution of income, disproportionally benefiting the lowest income countries (Figure 2). Similarly, using a discrete classification of countries according to income and malaria endemicity, greater average income gains would be achieved among poorer and higher endemic countries (Figures 3 and 4). These results are averages over the study period and may be different over different intervals of time (Table 4, column 6).

In terms of growth, our analysis on 5-year averages shows a negative and statistically significant effect of the lagged incidence of malaria on the subsequent growth rate of GDPpc PPP, with a 10% reduction in malaria incidence in period *p−*1 being associated with a GDPpc PPP growth of 0.1 percentage point higher in period *p* (Table 5, column 2). Restricting our sample to non-SSA countries yields similar results (Table 5, column 3). In contrast to the Gallup and Sachs study, we do not find evidence of an effect statistically distinguishable from zero of the log change in malaria incidence on economic growth in period *p.*

**Table 5:** Within regressions of annual GDPpc PPP growth rates on log malaria incidence and annual change in malaria incidence using 5-year averages

	All (1)	All (2)	Non-SSA (3)
Log malaria incidence_*p*−1_	−0.014*** [−0.023, −0.004]	−0.011** [−0.021, −0.001]	−0.010* [−0.020, 0.000]
Log change in malaria incidence_*p*_	−0.000 [−0.004, 0.004]	0.000 [−0.004, 0.004]	−0.004 [−0.012, 0.004]
Log GDPpc PPP_*p*−1_	−0.095*** [−0.140, −0.049]	−0.113*** [−0.162, −0.064]	−0.097*** [−0.147, −0.047]
Years of schooling _*p*−1_	0.007* [−0.001, 0.015]	0.000 [−0.008, 0.009]	−0.002 [−0.012, 0.009]
Life expectancy_*p*−1_	0.003* [−0.000, 0.006]	0.001 [−0.002, 0.004]	0.003 [−0.004, 0.009]
Trade per cent of GDP_*p*_	0.000 [−0.000, 0.000]	0.000 [−0.000, 0.000]	0.000 [−0.000, 0.000]
Rule of Law_*p*_	0.037* [−0.001, 0.075]	0.039** [0.003, 0.076]	0.035 [−0.012, 0.081]
Year effects	No	Yes	Yes
R-squared	0.256	0.287	0.205
Number of observations	270	270	208
Number of countries	135	135	104

Note:* *P* < 0.10, ** *P* < 0.05, *** *P* < 0.01. All estimations used standard errors clustered at the country level and 95% CIs are given in brackets below coefficients. The dependent variable is the 5-year average of instantaneous annual growth rate (*g*_*y*_) of GDPpc PPP:*g*_*y*_ = 1/*t*[ln(*Y*_*t*_)−ln(*Y*_*t*−1_)].

Next, we attempt to establish a causal relationship between malaria incidence and GDPpc PPP using insecticide resistance and antimalarial treatment failure as instruments. We find that confirmed mosquitos’ resistance status is significantly and positively associated with malaria case incidence (Table 6, Panel B). This result holds with and without the inclusion of year fixed effects and institutional- and trade-related covariates. Possible mosquitos’ resistance status is also positively correlated with malaria incidence but is significant only when the aforementioned covariates are included. The estimated second-stage coefficient of log malaria incidence on GDPpc PPP are negative, significant, and relatively stable (Table 6, Panel A, columns 1 and 2). Turning to our second IV candidate, antimalarial treatment failure, the first-stage coefficient is significant and negative, which is consistent with the observed higher levels of antimalarial treatment failure in lower malaria transmission areas.^55^ In high-transmission areas, such as SSA, an emerging drug-resistant parasite faces greater competition from drug-sensitive parasites that already occupy most of the host population, making them more prone to extinction. This is one explanation for the faster evolution of higher drug resistance in lower transmission settings.^55^ Again, the second-stage coefficients of log malaria incidence on GDPpc PPP are negative and significant (Table 6, Panel A, columns 3 and 4). The pooled-IV approach allows us to test for instrument coherence or exogeneity under the assumption that at least one instrument is valid. The Hansen *J*-statistic does *not* reject the null that insecticide and antimalarial drug resistance are valid instruments (Table 6, columns 1, 2, and 5). A note on instrument strength is, however, warranted despite the first-stage statistical significance of the instruments and *F*-statistics above the “rule of thumb” value of 10.^48^ Although all of our 2SLS estimations are robust to clustered standard errors at the subregional level (Table 6), they are not robust to the country-level clustering of standard errors (Table 7).

**Table 6:** Two-stage least squares regression of log GDPpc PPP on log malaria incidence—subregional level clustering of standard errors

Panel A: dependent variable—log GDPpc PPP					
	Panel IV (1)	Panel IV (2)	Panel IV (3)	Panel IV (4)	Pooled IV (5)
Log malaria incidence	−0.280* [−0.589, 0.030]	−0.291** [−0.572, −0.009]	−0.280* [−0.572, 0.013]	−0.358*** [−0.527, −0.190]	−0.208*** [−0.280, −0.137]
Rule of Law	–	0.070 [−0.055, 0.195]	–	0.143 [−0.035, 0.320]	0.272*** [0.167, 0.378]
Trade per cent of GDP	–	0.000 [−0.001, 0.001]	–	−0.001 [−0.004, 0.002]	−0.002 [−0.007, 0.003]
Year	Trend	Effects	Effects	Effects	Effects

Panel B: first-stage dependent variable—log malaria incidence					
Confirmed insecticide resistance	0.119** [0.002, 0.236]	0.130** [0.016, 0.243]	–	–	2.692*** [1.019, 4.365]
Possible insecticide resistance	0.086 [−0.046, 0.219]	0.093*** [0.061, 0.125]	–	–	–
Treatment failure (%)	–	–	−0.014*** [−0.021, −0.007]	−0.013*** [−0.017, −0.010]	0.004 [−0.291, 0.300]
Rule of Law	–	−0.014 [−0.168, 0.139]	–	−0.303* [−0.625, 0.018]	−1.268** [−2.427, −0.108]
Trade per cent of GDP	–	0.001 [−0.002, 0.004]	–	−0.004 [−0.013, 0.005]	0.001 [−0.031, 0.032]
Number of observations	462	431	196	186	131
Number of countries	66	63	49	45	31
First-stage *F*-statistic	27.21	43.27	20.59	23.39	78.96
Hansen *J*-statistic of overidentification	0.359	0.263	–	–	0.231

Note:*P < 0.10, **P < 0.05, ***P < 0.01. All panel estimations include country effects and all estimations used standard errors clustered at the subregional level; 95% CIs are given in brackets below coefficients.

**Table 7:** Two-stage least squares regression of log GDPpc PPP on log malaria incidence—country-level clustering of standard errors

Panel A: dependent variable—log GDPpc PPP					
	Panel IV (1)	Panel IV (2)	Panel IV (3)	Panel IV (4)	Pooled IV (5)
Log malaria incidence	−0.280 [−0.811, 0.252]	−0.291 [−0.775, 0.194]	−0.280* [−0.591, 0.031]	−0.358 [−0.850, 0.134]	−0.208*** [−0.363, −0.054]

Rule of Law	–	0.070 [−0.044, 0.183]	–	0.143 [−0.135, 0.420]	0.272 [−0.106, 0.650]
Trade per cent of GDP	–	0.000 [−0.002, 0.002]	–	−0.001 [−0.004, 0.002]	−0.002 [−0.006, 0.002]
Year	Trend	Effects	Effects	Effects	Effects

Panel B: first-stage dependent variable—log malaria incidence					
Confirmed insecticide resistance	0.119 [−0.039, 0.277]	0.130 [−0.047, 0.306]	–	–	2.771*** [1.042, 4.500]
Possible insecticide resistance	0.086 [−0.087, 0.260]	0.093 [−0.096, 0.282]	–	–	–
Treatment failure (%)	–	–	−0.013* [−0.028, 0.002]	−0.012 [−0.031, 0.008]	0.004 [−0.121, 0.129]
Rule of Law	–	−0.014 [−0.348, 0.320]	–	−0.292 [−0.931, 0.347]	−1.191* [−2.453, 0.072]
Trade per cent of GDP	–	0.001 [−0.004, 0.006]	–	−0.004 [−0.010, 0.003]	0.001 [−0.020, 0.022]
Number of observations	462	431	196	186	131
Number of countries	66	63	49	45	31
First-stage *F*-statistic	9.00	2.21	2.69	2.57	3.82
Hansen *J*-statistic of overidentification	0.032	0.015	–	–	0.609

Note:*P < 0.10, **P < 0.05, ***P < 0.01. All panel estimations include country effects and all estimations used standard errors clustered at the country level; 95% CIs are given in brackets below coefficients.

Finally, we present estimates of one potential channel through which malaria may affect economic outcomes—labor productivity. In Table 8, the coefficient on the interaction term of the labor share and malaria incidence is essentially a second derivative: the effect of a marginally higher labor share with a 10% increase in malaria incidence is negative and statistically significant with a magnitude of −1.1 (column 4). These estimations are relatively stable across specifications and control for a host of other confounding factors that may affect industry growth rates at the industry–year, country–year, and industry–country levels. Malaria elimination could increase, ceteris paribus, the effect of labor share on industry growth rates by 11 percentage points.

**Table 8:** Within regressions of log value added at the 2-digit industry level on log malaria incidence interacted with industry labor share

	(1)	(2)	(3)	(4)
Log value added_*t*−1_	−0.439*** [−0.489, −0.390]	−0.479*** [−0.532, −0.426]	−0.527*** [−0.590, −0.463]	−0.601*** [−0.671, −0.531]
Labor share	−1.546*** [−1.720, −1.371]	−1.548*** [−1.730, −1.367]	−1.810*** [−1.985, −1.635]	−1.822*** [−2.003, −1.642]
Log malaria incidence	0.052*** [0.013, 0.091]	0.037* [−0.004, 0.077]	–	–
Labor share* log malaria incidence	−0.174*** [−0.267, −0.081]	−0.181*** [−0.274, −0.088]	−0.107** [−0.197, −0.017]	−0.111** [−0.201, −0.022]
Country–year effects	No	No	Yes	Yes
Industry–year effects	No	Yes	No	Yes
R-squared	0.440	0.505	0.553	0.628
Number of observations	11,606	11,606	11,606	11,606
Number of country–industry pairs	1,597	1,597	1,597	1,597

Note:∗*P* < 0.10,∗∗*P* < 0.05,∗∗∗*P* < 0.01. Labor share is defined as the share of wages in the total value added. Column (1) includes individual year effects and all estimations include country-industry fixed effects. Standard errors are clustered at the country–industry level; 95% CIs are given in brackets next to coefficients.

## DISCUSSION

This study took a contemporary look at the seminal work by Gallup and Sachs published in 2001, which still shapes the global advocacy agenda with regard to malaria and its association with economic development. We found that in OLS specifications similar to the Gallup and Sachs study, a 10% reduction in malaria incidence is associated with GDPpc PPP levels 1.8–1.9% higher over the period 2000–2017. In our preferred specification, which controls for country-specific effects, such as institutions, socioeconomic, and geographical characteristics, as well as year effects, reducing malaria incidence by 10% was associated with an increase in GDPpc PPP of nearly 0.3% on average over the study period. In terms of growth, we found that a 1% reduction in malaria incidence in the previous period is associated with a 0.01 percentage point faster growth in GDPpc PPP in the current period. Reduction in incidence would disproportionately benefit countries with the highest malaria burden and lowest income levels. Unlike Gallup and Sachs, we did not find evidence of a significant relationship between change in malaria incidence and income growth rates using consecutive 5-year periods.^14^ Overall, although our numbers are substantial, they are much smaller than those reported in the original work.^14^ This is probably inevitable in that the marginal benefits to malaria control have been reduced by concerted international action over the past 17 years.^27^ These efforts have already led to substantial dividends as a consequence of reductions in malaria incidence since 2000. Most likely, the differences in results reflect differences in the methods and data that were used. A significant slightly smaller negative relationship between malaria incidence and per capita income growth for the period 1983–1997 was also reported elsewhere.^56^

Our attempt to establish a causal relationship between malaria incidence and income using 2SLS has several caveats that need to be discussed. First, the analyses are performed on a subsample of countries and years for which antimalarial treatment failure and insecticide resistance data are available. Although the studies from which the data are collected span several years and multiple studies were available each year, the data had to be collapsed to match with corresponding GDPpc PPP and covariates data at the country–year level. Second, clustering of standard errors is usually recommended at the level of exposure, which, in our case, is antimalarial treatment failure and resistance of mosquitoes to insecticides. Our strongest results come from analyses where the standard errors are clustered at the subregional level. This approach lies on the assumption that insecticide resistance and antimalarial treatment failure may emerge because of factors that are consistent more within subregions than arbitrary country borders, such as environmental and biological factors. Under this assumption, our instruments seem to be valid under both panel IV and pooled-IV methods. Our panel and pooled-IV estimations are, however, not robust to clustering at the country level, which may be due to insufficient observations for each country or insufficient variation within the data. Third, the estimates from an IV approach may, of course, not converge to the true parameter value but instead give a local impact driven by variations in the specific IV, also known as the LATE.^57,58^ Fourth, the use of resistance of mosquitoes to pyrethroid-only–based insecticides as instrument may overlook the relationship between insecticide resistance, agricultural practices, and economic outcomes, thus challenging the exclusion restriction.^59,60,61^

Finally, our examination of one potential mechanism, labor productivity, shows that ceteris paribus, industries with the same level of labor intensity, given by the share of wages in total value added, tend to grow slower in countries with higher malaria. Our findings are comparable with recent estimates from other empirical studies which use micro-level data to examine the link between malaria and development outcomes, relying on some exogenous variation to attribute causality. A study by Bleakley published in 2010 identifies the effect of childhood exposure to malaria elimination efforts on subsequent adult labor productivity by comparing cohorts based on birth years before and after elimination efforts as well as across regions with high and low malaria prevalence.^34^ Compared with non–malaria-endemic areas, cohorts of children born after elimination efforts had higher income as adults than the preceding generation.^34^ Persistent childhood infection of malaria was found to reduce adult income by 50% with similar estimates obtained in separate analyses for the United States, Mexico, Brazil, and Colombia.^34^ A similar identification strategy applied to India finds that a 10% point decrease in malaria incidence raises per capita expenditure by 1.5–6.8%.^32^ In Uganda, findings suggest that malaria elimination would lead to 5–20% increase in income annually via improvements in educational attainment.^33^

The more recent empirical literature on health and economic development has shown that the link between the two is relatively tenuous.^3,9,12^ This is in part due to life expectancy at birth, the most commonly used measure of health outcomes, being jointly determined with income and growth. Moreover, it is often the case that the disease burden, particularly in tropical areas, impacts economic outcomes via other channels such as the acquisition of human or physical capital, greater fertility, etc. Our malaria-related case study contributes to this literature on the intrinsic link between health and economic growth and underscores the continued relevance and important role of malaria control in the current agenda for sustainable development.
